# Guideline development in harm reduction: Considerations around the meaningful involvement of people who access services

**DOI:** 10.1016/j.dadr.2022.100086

**Published:** 2022-08-12

**Authors:** Alison Adams, Max Ferguson, Alissa M. Greer, Charlene Burmeister, Kurt Lock, Jenny McDougall, Marnie Scow, Jane A. Buxton

**Affiliations:** a British Columbia Center for Disease Control, 655W 12th Avenue, Vancouver, BC V5Z 4R4, Canada; b School of Population and Public Health, University of British Columbia, 2206 E Mall, Vancouver, BC V6T 1Z8, Canada; c School of Criminology, Simon Fraser University, 8888 University Drive, Burnaby, BC V5A 1S6, Canada

**Keywords:** Guidelines, Harm reduction, Public health, Peer engagement

## Abstract

**Background:**

Harm reduction seeks to minimizes the negative effects of drug use while respecting the rights of people with lived and living experience of substance use (PWLLE). Guideline standards (“guidelines for guidelines”) provide direction on developing healthcare guidelines. To identify essential considerations for guideline development within harm reduction, we examined whether guideline standards are consistent with a harm reduction approach in their recommendations on involving people who access services.

**Methods:**

We searched the literature from 2011–2021 to identify guideline standards used in harm reduction and publications on involving PWLLE in developing harm reduction services. We used thematic analysis to compare their guidance on involving people who access services. Findings were validated with two organizations of PWLLE.

**Results:**

Six guideline standards and 18 publications met inclusion criteria. We identified three themes related to involving people who access services: *Reasons for Involvement, Methods of Involvement*, and *Factors in Success*. Subthemes varied across the literature. We identified five essential considerations for guideline development in harm reduction: establishing a shared understanding of reasons for involving PWLLE; respecting their expertise; partnering with PWLLE to ensure appropriate engagement; incorporating perspectives of populations disproportionately affected by substance use; and securing resources.

**Conclusion:**

Guideline standards and the harm reduction literature approach the involvement of people who access services from different perspectives. Thoughtful integration of the two paradigms can improve guidelines while empowering PWLLE. Our findings can support the development of high-quality guidelines that align with the fundamental principles of harm reduction in their involvement of PWLLE.

## Highlights

• Guideline developers in harm reduction should begin by engaging consumers.
• Developers would benefit from co-production of standards for consumer involvement.
• Supporting peer organizations facilitates community engagement.
• Resources and organizational support are critical to meaningful involvement.

## 1 Introduction

A guideline is an evidence-based document that includes recommendations on “whether, when and how to undertake specific actions such as clinical interventions, diagnostic tests or public health measures” (World Health Organization, 2021, para. 2). Guidelines are intended to improve the quality of care and limit unintended variation in practice (Rosenfeld et al., 2013). Guideline standards, or “guidelines for guidelines”, further support these aims by providing practical direction on the principles and methods of guideline development in health care. A recent systematic review searched for guideline standards created by institutions that develop clinical guidelines and identified over 50 documents published since 2003 by medical associations, public institutions, and other organizations (Selva et al., 2017).

Guideline standards, though often created with clinical practice in mind, have also been used to develop guidelines in public health. Applying guideline standards in public health presents numerous challenges, which have been discussed most extensively in relation to the highly influential GRADE approach to guideline development. Difficulties include the incompatibility of complex public health interventions and randomized controlled trials, resulting in a lack of high-quality evidence to inform guidelines, and the difficulty of incorporating non-epidemiological evidence relating to contextual factors that affect interventions (European Centre for Disease Prevention and Control [ECDC], 2011; Hilton Boon et al., 2021; Montgomery et al., 2019; Rehfuess and Akl, 2013).

Individuals developing guidelines for harm reduction services may also question whether existing guideline standards are consistent with the fundamental principles of harm reduction in their approach to involving people who access services. Harm reduction is most commonly discussed in the context of drug use, where it has been described as “policies, programmes and practices that aim to minimise negative health, social and legal impacts associated with drug use, drug policies and drug laws.” (Harm Reduction International, 2022). The approach recognizes that many health and social problems associated with drug use are caused or exacerbated by the marginalization of people with lived and living experience of substance use (generally abbreviated as “PWLLE”, with the truncation serving as an acknowledgement of the fact that these individuals also bring experience from other areas of life).^1^ Respect for the rights and preferences of PWLLE is a core facet of the harm reduction ethos (Riley and Pates, 2012). As such, involving PWLLE in program and policy development is considered critical to ensuring high-quality harm reduction services (BC Centre for Disease Control, 2018).

In this article, we describe how guideline standards differ from the harm reduction literature in their involvement of people who access services and explore the implications of these differences. Our aim was to identify essential considerations for guideline developers working in harm reduction in order to support the development of high-quality guidelines that align with the fundamental principles of harm reduction in their approach to involving people who access services.

## 2 Methods

A member of the research team searched the peer-reviewed and grey literature to identify 1) guideline standards used in harm reduction and 2) publications with guidance on involving people who access services in developing harm reduction services. To ensure retrieval of contemporary material reflecting a recent shift towards increased involvement of people who access services (Chachoua et al., 2020), we limited our review to documents published in the preceding 10 years (2011–2021; see Appendices A–D for exact search dates). The search was also limited to English-language publications. We used thematic analysis (Braun and Clarke, 2006) to compare and contrast the two bodies of literature. Screening, coding, and a preliminary thematic framework were completed by one author and validated with co-authors. Preliminary findings were reviewed with two organizations of PWLLE to verify the credibility of the analysis and identify themes that merited deeper exploration. Their insights were invaluable in guiding the final analysis and informed the identification of five essential considerations for guideline developers working in harm reduction. We sought additional feedback from one of the organizations following the development of these points, and subsequently re-worded and expanded several points to ensure appropriate terminology and relevance to the needs of PWLLE.

### 2.1 Identification of guideline standards

**Fig. 1:**
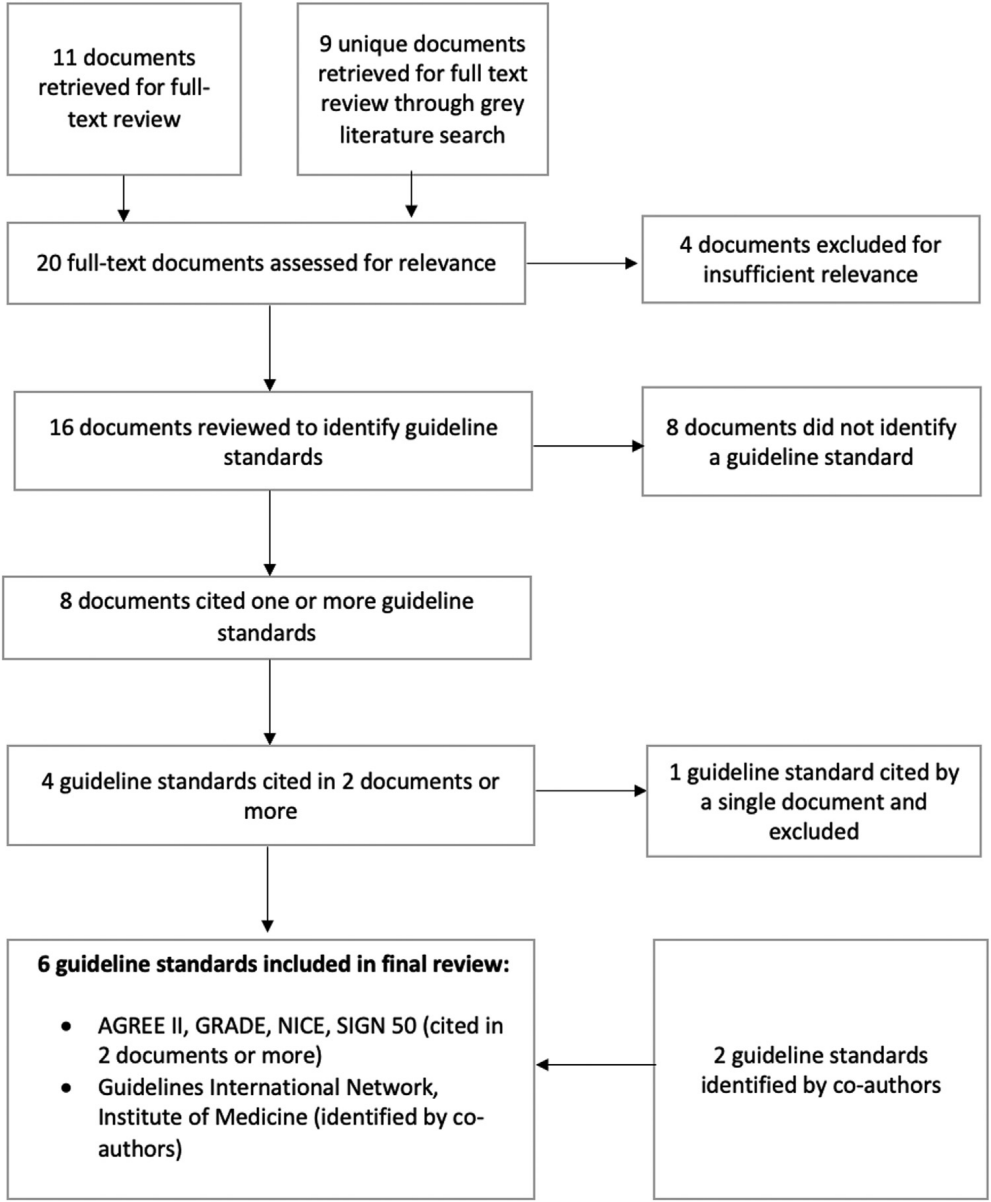
Flow chart for identification of guideline standards used in harm reduction.

To identify guideline standards used in harm reduction, we searched the peer-reviewed and grey literature for harm reduction guidelines and accounts of their development and extracted information on the standard(s) used. See Fig. 1 for a study flow diagram. To identify peer-reviewed literature, we searched Ovid Medline using keywords and controlled vocabulary from the MeSH (Medical Subject Headings) thesaurus. See Appendix A for details. We searched the grey literature using the *Grey Matters* checklist developed by the Canadian Agency for Drugs and Technologies in Health (2019). Websites listed under “Clinical Practice Guidelines” were searched using keywords related to harm reduction. In addition, we searched Google and Google Scholar and reviewed the first five pages of results for relevant material. See Appendix B for details.

The Ovid Medline search retrieved 241 citations. We screened titles and abstracts to identify guidelines for harm reduction services and accounts of their development. See Table 1 for inclusion and exclusion criteria. After title and abstract screening, we identified 11 documents as potentially relevant and retrieved the full text for review. We screened the grey literature using the same inclusion and exclusion criteria and identified an additional nine unique documents. No additional documents were identified through citation chaining. After full-text review, four of the 21 documents were excluded for insufficient relevance. Of the 16 remaining documents, eight did not reference any guideline standards. The remaining eight documents identified one or more guideline standards. See Supplementary Table 1 for details. These included the following:

• *The AGREE II Instrument* (3 documents) (AGREE Next Steps Consortium, 2017)
• *GRADE Handbook* (3 documents) (Schünemann et al., 2013).
• *Developing NICE guidelines: the Manual* (2 documents) (NICE, 2020)
• *SIGN 50: A Guideline Developer's Handbook* (2 documents) (SIGN, 2019a)
• *Canadian Task Force on Preventive Health Care* [CTFPHC] (1 document) (CTFPHC, 2014)

**Table 1:** Inclusion and exclusion criteria.

	**Inclusion criteria**	**Exclusion criteria**
Guideline standards:	The document is a guideline or an account of a guideline's development;The guideline relates to harm reduction services in the context of substance use (e.g., take-home naloxone programs, supervised consumption services).	Documents other than guidelines and accounts of guideline development; Guidelines related to harm reduction in the context of gambling or tobacco use; Guidelines on opioids for pain management.
Harm reduction publications:	The document has substantive content on involving PWLLE in developing harm reduction services; The document is relevant to the development of harm reduction services in the context of substance use (e.g., take-home naloxone programs, supervised consumption services).	Documents focusing exclusively or primarily on involving PWLLE in service delivery (e.g., as employees) or research; Documents focusing on harm reduction in the context of gambling or tobacco use.

Standards referenced in two or more documents were included in this review because of their established history of use in harm reduction. Co-authors identified two additional standards as relevant within the field of harm reduction:

• The Institute of Medicine's *Clinical Practice Guidelines We Can Trust* (IoM et al., 2011)
• The Guidelines International Network (GIN) standards (Qaseem et al., 2012)

Review of several reports on guideline development indicated that these standards were in widespread use (ECDC, 2011; Rosenfeld et al., 2013; Schünemann et al., 2006). Therefore, a total of six guideline standards were included in this review. See Supplementary Table 2 for additional details.

### 2.2 Identification of harm reduction publications

**Fig. 2:**
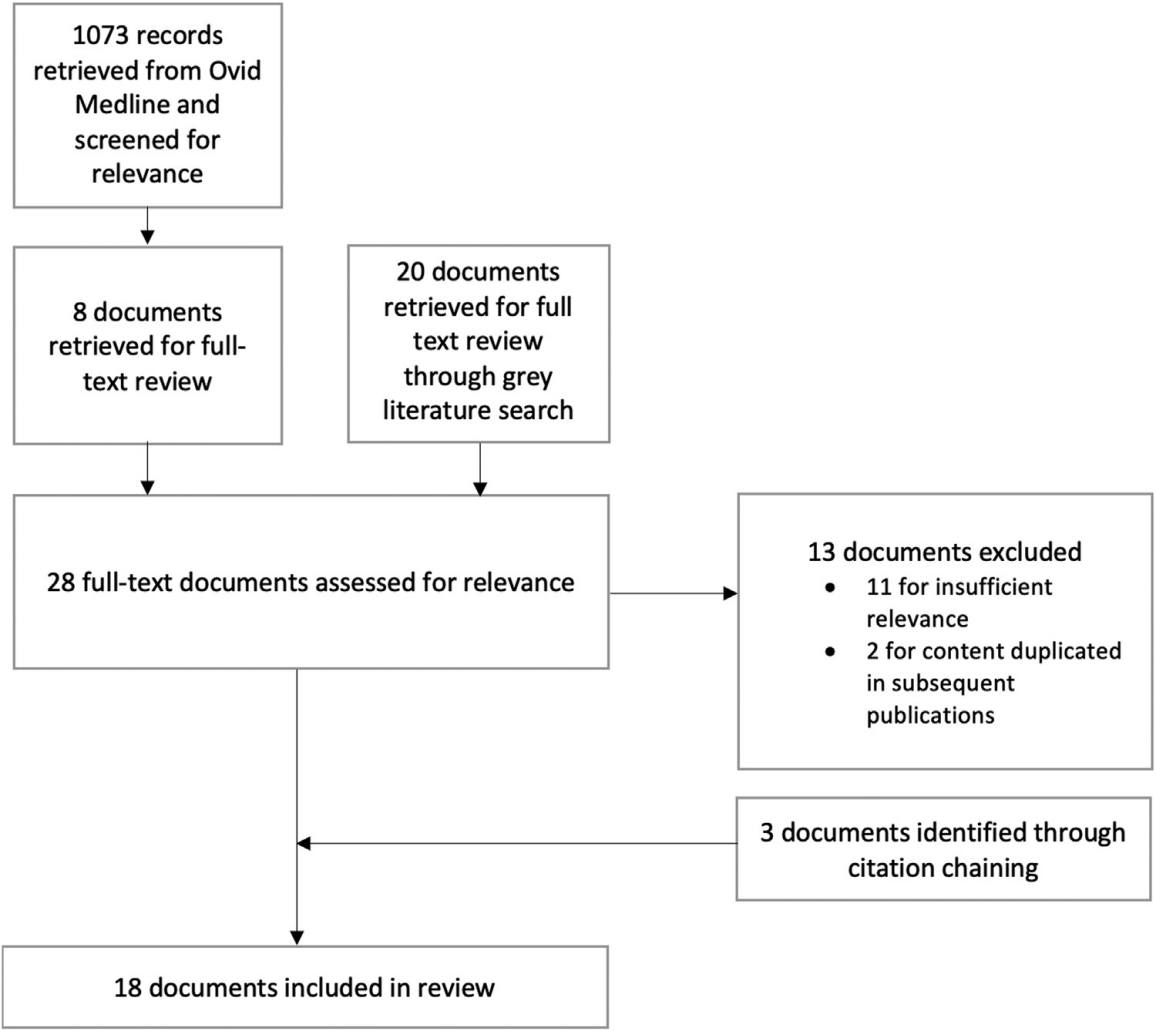
Flow chart for identification of harm reduction publications.

We searched the peer-reviewed and grey literature for publications on involving PWLLE in the development of harm reduction services. See Fig. 2 for a study flow diagram. To identify peer-reviewed literature, we searched Ovid Medline using keywords and controlled vocabulary from the MeSH (Medical Subject Headings) thesaurus. Subject matter experts provided input to ensure that the search strategy captured international variation in terms for PWLLE, including “peers”, “consumers”, “service users”, and “people who use drugs”. See Appendix C for additional details. To identify grey literature, we searched the websites of harm reduction organizations, relevant branches of government, and associations of PWLLE. We used browsing and keyword searching to identify relevant documents. We also searched Google and Google Scholar and reviewed the first five pages of results. In addition, we solicited recommendations from experts in harm reduction. See Appendix D for additional details.

The Ovid Medline search retrieved 1073 citations. We screened titles and abstracts to identify documents on involving PWLLE in the development of harm reduction services. See Table 1 for inclusion and exclusion criteria. After title and abstract screening, eight documents from the Ovid Medline search were identified as potentially relevant and retrieved for full-text review. We screened the grey literature against the same inclusion and exclusion criteria and identified an additional 20 documents. After full-text review, 11 documents were excluded for insufficient relevance. Another two were excluded because their content was incorporated into a longer and more detailed subsequent publication (Greer et al., 2017; Capitalized 'BC Centre for Disease Control'.). The references of the remaining 15 documents were scanned to identify additional articles meeting inclusion criteria. Three additional documents were identified. Therefore, a total of 18 documents on involving PWLLE in the development of harm reduction services were included in this review. Three of the documents were identified in the search of peer-reviewed literature and 15 in the grey literature search. See Table 2 for a geographical breakdown of the literature.

**Table 2:** Geographical breakdown of harm reduction publications.

	No. of documents	Citations
Canada	9	Alberta Health Services, 2018; Belle-Isle et al., 2016; Canadian AIDS Society, 2015; Canadian Association of People Who Use Drugs, 2014; Canadian Centre on Substance Use and Addiction [CCSA], 2021; Giacomazzo, 2021; Greer et al., 2016, 2017, 2019
Australia	4	Advocacy Tasmania, 2011; Australian Injecting and Illicit Drug Users League [AIVL], 2012; Health Consumers Council Western Australia, 2016; New South Wales Ministry of Health, 2019
The Netherlands	2	Kools, 2013; Schiffer and Correlation-European Harm Reduction Network [C-EHRN], 2021
Wales	1	Welsh Government, 2014
International or unspecified	2	Ti et al., 2012; United Nations Office on Drugs and Crime et al., 2017

Although all included documents met our inclusion criteria, they were heterogeneous in their aims and authorship. Some provided direction for specific organizations or branches of government; others outlined general principles. See Supplementary Table 3 for additional details. The documents were produced with various levels of involvement of PWLLE.

### 2.3 Analysis

We used thematic analysis (Braun and Clarke, 2006) to compare the guideline standards and the harm reduction documents. First, a member of the research team reviewed the included documents to identify and code relevant sections of the text. For the guideline standards, content was considered relevant if it discussed involving people who access services in guideline development. Broad discussions of “major stakeholders”, “individuals with relevant expertise”, etc., were not considered relevant unless the guideline standard specified that this group included people who access services. Introductory material, acknowledgements, references, and appendices were not reviewed. For the harm reduction documents, content was considered relevant if it discussed involving PWLLE in developing harm reduction services. Content on employing or conducting research with PWLLE was not considered relevant.

After relevant content had been identified, inductive coding was used to describe discrete segments of meaningful text. Coding was performed using Microsoft Word and Taguette, an open-source software for qualitative coding (Rampin et al., 2021). Documents were read multiple times and codes were refined repeatedly to reflect patterns of meaning within the data set. The codes formed the basis for a framework of themes and subthemes capturing the range and diversity of content on involving people who access services. To illustrate, the following section of text was initially coded as *Transportation needs:*

*If people who use drugs are travelling to attend your meeting, they may require identification documents. They may require support to obtain those prior to a meeting.* (Belle-Isle et al., 2016, p. 5).

It subsequently became clear that there were conceptual links between *Transportation needs* and various other codes, including *Modes of communication, Access to substances,* and *Childcare.* In the final thematic framework, these codes were merged under the subtheme *Logistics,* which itself was positioned within the broader theme *Factors in Success.* We developed a codebook with definitions and examples of each theme and subtheme to ensure consistency throughout the analysis (Supplementary Table 4). Next, we compared the themes and subthemes found within the guideline standards and the harm reduction documents. We noted the distribution of subthemes and described variation in the content of the subthemes within each body of literature.

After completing a preliminary analysis, we reviewed findings with two organizations of PWLLE that provide consultation to researchers and government bodies: Professionals for Ethical Engagement of Peers (https://towardtheheart.com/peep) and the Peer2Peer (P2P) project group (https://towardtheheart.com/peer2peer-project). Their input guided the interpretation of findings and the development of five essential considerations for guideline developers in harm reduction.

## 3 Results

Our thematic analysis identified three themes that spanned both bodies of literature: *Reasons for Involvement, Methods of Involvement,* and *Factors in Success.* Each theme contained numerous subthemes.

### 3.1 Reasons for involvement

The theme *Reasons for Involvement* included content that addressed the question “Why should people who access services be involved?” Nine subthemes were identified. See Table 3 for the distribution of subthemes.

**Table 3:** Distribution of subthemes within Theme 1: reasons for involvement. 1.

Subtheme	Harm Reduction Documents																		Guideline Standards					
	1	2	3	4	5	6	7	8	9	10	11	12	13	14	15	16	17	18	1	2	3	4	5	6
Quality	x	x	x	x	x	x	x				x		x	x	x	x		x		x		x	x	x
Acceptability	x						x	x	x	x	x			x	x	x	x	x		x		x	x	
Equity			x	x	x	x	x		x	x	x	x		x		x	x	x		x		x		
Human rights	x		x			x	x	x	x		x	x	x		x	x	x	x						
Value to participants	x	x	x	x			x	x	x		x		x	x		x	x	x						
Scope	x			x	x	x	x	x	x	x	x		x	x	x	x			x	x	x	x	x	
Recommendations																			x	x		x	x	x
Credibility	x		x					x			x							x					x	
Cost-effectiveness	x										x							x						

1 See Supplementary Table 5 for key to numerical references.

#### 3.1.1 Scope, recommendations, quality, acceptability, equity, credibility

The guideline standards viewed involvement of people who access services as valuable in defining guideline scope (5/6 guideline standards), formulating recommendations (5/6), and improving the quality of the final product (4/6). A smaller number stated that involving people who access services would improve the acceptability (3/6) and credibility (1/6) of guidelines and enhance equity (2/6). The harm reduction documents described many of the same benefits in a more general context. Involvement of PWLLE was seen as helpful in defining the scope of programs and policies (13/18 harm reduction documents) and increasing their quality, (13/18), as well as improving equity (13/18), acceptability (11/18), and credibility (5/18).

Harm reduction documents that identified increased quality as a benefit emphasized that PWLLE were experts in their own right, able to share knowledge and perspectives that could ensure program relevance, identify threats to program success, and highlight solutions that might otherwise be overlooked. the Canadian Association of People Who Use Drugs (2014), for instance, stated that PWLLE “have unique insights into what reforms will actually contribute to the health, dignity and self-determination for people who use drugs” (p. 5).

Health equity was identified as a reason for involvement in guideline standards and the harm reduction documents However, the relationship between equity and the involvement of people who access services was conceptualized differently in the two bodies of literature. When equity was mentioned in the guideline standards, it was as a possible downstream effect of a guideline. The harm reduction literature, in contrast, described increased health equity as a benefit of the involvement process itself.

#### 3.1.2 Value to participants, human rights, cost-effectiveness

Three subthemes appeared in only the harm reduction documents: *Value to Participants* (13/18 harm reduction documents), *Human Rights* (13/18), and *Cost-Effectiveness* (3/18). *Value to Participants* included content describing benefits of PWLLE involvement for participants. These benefits included empowerment, new skills and knowledge, and social benefits (e.g., destigmatization; social inclusion). A majority of the harm reduction documents also made a case for involvement from a human rights perspective, arguing that PWLLE have “an ethical and imperative right to be involved in the decisions affecting their lives” (Ti et al., 2012, p. 9). Two harm reduction documents suggested that involving PWLLE could reduce costs by eliminating ineffective services (Advocacy Tasmania, 2011) and improving program uptake (Greer et al., 2017), while a third asserted that it was cost-effective (Schiffer & Correlation-European Harm Reduction Network [C-EHRN], 2021).

### 3.2 Methods of involvement

The second theme, *Methods of Involvement,* included content that addressed the question “How should people who access services be involved?”. Nine subthemes were identified. See Table 4 for the distribution of subthemes.

**Table 4:** Distribution of subthemes within Theme 2: Methods of Involvement.^1^

Subtheme	Harm Reduction Documents																		Guideline Standards					
	1	2	3	4	5	6	7	8	9	10	11	12	13	14	15	16	17	18	1	2	3	4	5	6
Leveraging & supporting communities of people who access services	x	x	x		x		x			x	x	x	x	x	x	x	x							
Committee membership		x	x	x	x							x		x	x	x		x		x	x	x	x	x
Interviews & focus groups	x	x		x	x		x					x		x		x				x	x	x		
Public forums					x		x							x	x	x				x		x	x	
Consultation	x	x												x	x	x		x	x	x	x	x	x	
Review of drafts														x		x				x	x	x	x	x
Literature review																			x	x	x	x	x	
Engagement at point of care	x																		x	x		x	x	
Clinicians as representatives of people who access services		x																	x				x	

1 See Supplementary Table 5 for key to numerical references.

#### 3.2.1 Circulating drafts for review, interviews & focus groups, public forums, committee membership, consultation

Guideline standards were more likely to suggest circulating drafts for review (5/6 guideline standards versus 2/18 harm reduction documents), conducting interviews and focus groups with people who access services (3/6 guideline standards versus 8/18 harm reduction documents), and holding public forums (3/6 guideline standards versus 5/18 harm reduction documents). Committee membership, including representation of people who access services on advisory committees or in the guideline development group, was also mentioned more frequently in the guideline standards (5/6 guideline standards versus 9/18 harm reduction documents). However, several guideline standards suggested “patient representatives” or “lay members”, which may include carers or members of the general public (IoM et al., 2011; NICE, 2020; SIGN, 2019a). Statements that people who access services should be involved through “consultation” were more common in the guideline standards (5/6 guideline standards versus 5/18 harm reduction documents).

#### 3.2.2 Leveraging & supporting communities of people who access services

Most harm reduction documents (13/18) observed that existing communities of PWLLE could facilitate communication with people who access services and enhance the quality of engagement. Supplying these communities with funding and other resources was seen as a way of supporting involvement. This subtheme was not found in the guideline standards.

#### 3.2.3 Literature review, engagement at point of care, clinicians as representatives of people who access services

Three themes described forms of indirect involvement and were predominantly seen in the guideline standards. Most suggested conducting literature reviews as a method for determining the values and preferences of people who access services (5/6 guideline standards). Four of six standards stated that guidelines should support shared decision-making between providers and people who access services at the point of care. A smaller proportion (2/6) stated that clinicians and other guideline developers could act as proxies for people who access services when necessary, using their clinical experience to identify values and preferences. However, this method was recognized as suboptimal (IoM et al., 2011; Schünemann et al., 2013).

### 3.3 Factors in success

The third theme, *Factors in Success,* included content that addressed the question “What factors have an impact on the success (or failure) of involving people who access services?” Thirteen subthemes were identified. See Table 5 for the distribution of subthemes.

**Table 5:** Distribution of subthemes within Theme 3: factors in Success.^1^

Subtheme	Harm Reduction Documents																		Guideline Standards					
	1	2	3	4	5	6	7	8	9	10	11	12	13	14	15	16	17	18	1	2	3	4	5	6
Resources	x	x	x	x	x	x	x		x	x	x	x	x		x	x		x				x	x	
Logistics		x		x	x		x		x		x	x				x								
Lack of research	x			x					x	x	x				x			x		x			x	
Representation	x	x	x	x		x	x		x	x	x	x	x	x		x		x	x	x		x	x	
Skills & literacy	x	x	x		x			x		x	x	x	x			x		x				x	x	x
Transparency	x	x	x	x	x		x			x	x	x	x	x		x		x	x	x	x	x	x	x
Trust & power	x	x	x	x	x	x	x	x	x	x	x	x	x	x	x	x	x	x						
Compensation	x	x	x	x			x	x	x		x	x				x		x						
Stigma		x	x	x	x		x	x	x	x	x	x	x		x	x		x						
Participant capacity	x	x	x	x	x	x	x	x	x	x	x	x					x	x						
Tokenism		x	x		x	x	x	x	x	x	x	x	x	x		x	x							
Social support	x	x	x		x		x		x		x	x				x	x	x						
Systemic factors	x	x	x				x	x	x		x	x			x	x	x	x						

1 See Supplementary Table 5 for key to numerical references.

#### 3.3.1 Representation

The challenge of selecting a small number of individuals to represent the views of a large and heterogeneous population was discussed in the guideline standards (4/6) and the harm reduction documents (14/18). In the harm reduction documents, people developing services were encouraged to seek out participants with diverse experiences. These included people representing various patterns of drug use (7/18), Indigenous people (5/18), pregnant women (3/18), and people of assorted racial and/or cultural backgrounds, age groups, genders, and sexual orientations. The guideline standards also recognized many subpopulations with distinct concerns, but tended to place less emphasis on direct representation as a means of incorporating their perspectives.

#### 3.3.2 Skills & scientific literacy

The need for participants to have a certain level of scientific literacy and an understanding of policy development was discussed in the guideline standards (3/6) and the harm reduction documents (11/18). All stated that training should be offered when necessary. However, differences in the way this issue was framed suggested that the appropriate role of people who access services was viewed differently in the two bodies of literature. For instance, one guideline standard identified “the ability to examine evidence and recommendations dispassionately” as a critical component of scientific literacy and characterized personal experience with a condition or an advocacy role as potential threats to this ability (IoM et al., 2011, p. 90). The harm reduction documents, in contrast, treated personal experience as a form of expertise and included a description of advocacy as a responsibility of organizations of PWLLE: “DUOs [drug user organizations] often have to represent complex issues, with a responsibility to act as powerful advocates on behalf of their constituents, without being characterised as ‘difficult’ or unwilling to compromise, or seen as unrealistic about political imperatives” (Australian Injecting & Illicit Drug Users League [AIVL], 2012, p. 42).

#### 3.3.3 Resources

Resources were discussed more frequently in the harm reduction documents (2/6 guideline standards versus 15/18 harm reduction documents). In the guideline standards, resources were briefly mentioned as a practical limitation to involving people who access services. The harm reduction documents explored the topic more extensively, and often described the resources required to involve PWLLE in a meaningful way (e.g., sufficient time, with early engagement of PWLLE; secure funding; preparation of project staff; training materials).

#### 3.3.4 Transparency

The subtheme *Transparency* was found in all the guideline standards (6/6) and many of the harm reduction documents (13/18). Guideline standards tended to focus on the importance of documenting the involvement of people who access services and the outcomes of their involvement. Some standards provided detailed guidance on how to report this process (e.g., AGREE Next Steps Consortium, 2017). The harm reduction literature placed greater emphasis on the need for transparency in interactions with PWLLE participants, particularly around the objectives of involving PWLLE and the intended level of engagement. Transparency was linked to accountability in both bodies of literature. In the harm reduction documents, it was also presented as a guard against tokenism.

#### 3.3.5 Lack of research

A lack of research on methods of involvement, leading to uncertainty around how to involve people who access services in a systematic and effective way, was identified as a problem in both the guideline standards (2/6) and the harm reduction documents (7/18). Within this group of publications, there was general consensus on the need for additional research to support evidence-based guidance and frameworks for engagement.

#### 3.3.6 Trust & power, stigma, compensation, systemic factors, tokenism, participant capacity, social support, logistics

The subthemes *Trust & Power, Stigma, Compensation, Systemic Factors, Tokenism, Participant Capacity, Social Support*, and *Logistics* were found in only the harm reduction documents. All documents (18/18) discussed power differentials when involving PWLLE and the mitigating effects of mutual trust and respect. Stigma, perpetuated in part by the criminalization of substance use (Giacomazzo, 2021; Greer et al., 2019; Health Consumers Council Western Australia, 2016; Schiffer and C-EHRN, 2021; Ti et al., 2012) was described as a major barrier to positive working relationships in a large proportion of the documents (14/18). Many of these documents also identified discrimination as a barrier. It was observed that stigma could cause discrimination and that discrimination could result in stigma (CCSA, 2021), suggesting a self-perpetuating cycle.

The subthemes *Compensation* (11/18) and *Systemic Factors* (11/18) were closely linked to these social dynamics. Compensation was described as both a reflection and a determinant of power relations. Providing compensation for PWLLE demonstrated recognition of their expertise and respect for their time (CCSA, 2021; Greer et al., 2017, 2019; Health Consumers Council Western Australia, 2016). Conversely, neglecting to offer appropriate compensation was perceived as stigmatizing, inequitable, or disrespectful (CCSA, 2021; Greer et al., 2017; Schiffer and C-EHRN, 2021). Systemic factors, including organizational culture, political support, and the media's portrayal of PWLLE, were identified as critical determinants of the success of involving PWLLE. Unsupportive institutions were perceived as one of many factors contributing to the risk of *Tokenism* (14/18), meaning involvement without the intention and/or ability to transfer any power to people who access services. Tokenism was often contrasted with “meaningful” or “authentic” engagement, which were built on respectful relationships between participants and required genuine intention to share decision-making power with PWLLE.

The subtheme *Participant Capacity* (13/18 documents) was used for content acknowledging the emotional burden of involvement for PWLLE and the effect of personal circumstances on individual ability to participate in the process. A related subtheme was *Social Support* (11/18), which was used for passages identifying the value of peer support and mentorship for PWLLE participating in service development. Several harm reduction documents recommended that service developers partner with groups of PWLLE, or, at a minimum, two people (Alberta Health Services, 2018; Canadian AIDS Society, 2015; CCSA, 2021; Greer et al., 2017).

The subtheme *Logistics* (8/18 documents) included content on practical considerations for ensuring that activities related to service development were accessible to PWLLE. Suggestions included holding meetings in locations that could be reached by public transit (CCSA, 2021) and providing harm reduction supplies at meetings that included PWLLE (Belle-Isle et al., 2016).

## 4 Discussion

**Fig. 3:**
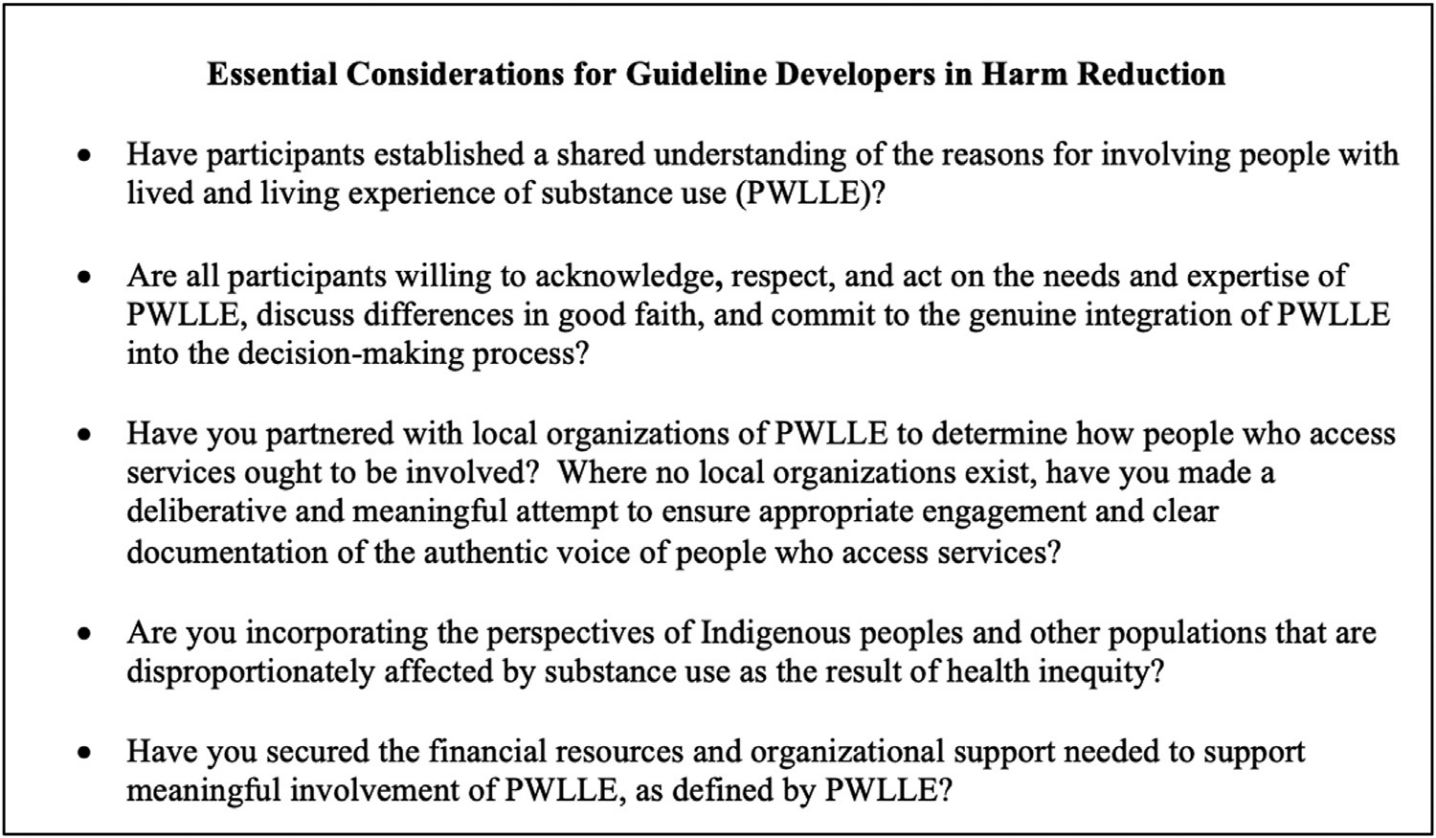
Essential considerations for guideline developers working in harm reduction.

We used thematic analysis to describe how guideline standards differ from the harm reduction literature in their discussion of involving people who access services. We examine the implications of these differences below. Based on our analysis, we identified five essential considerations for guideline developers aiming to create high-quality guidelines for harm reduction services while involving people who access services in a way that is consistent with the fundamental principles of harm reduction (Fig. 3).

### 4.1 Conceptual basis for involvement

The guideline standards and the harm reduction documents tended to present different reasons for involving people who access services. Guideline standards supported involvement on the grounds of its impact on guideline quality and focused on the role of the values and preferences of people who access services in defining scope and developing recommendations. These findings are consistent with Legare et al. (2011), who conducted a knowledge synthesis on patient and public involvement in guideline development. The harm reduction documents, while recognizing these benefits, also advocated for involvement of people who access services as a means of increasing health equity, empowering participants, and satisfying an ethical imperative.

Several distinct discourses underlying support for involving people who access services in policy development have been recognized in the literature. The harm reduction literature generally reflects “the democratic model” (Wait and Nolte, 2006), in which involvement is justified on the basis of the public's right to participate in political processes. Flood (2015), writing in support of this view, states that involving people who access services reflects the principles of natural justice: “public participation is inherently valuable even if one can't immediately discern an impact upon policy [. . .] people are entitled to a fair hearing when decisions are being made that impact them.” (p. 382). Under this model, a key function of involvement is the conferral of legitimacy on the resulting policy or program (Flood, 2015). Involvement of people who access services is also seen as having the potential to improve equity, although this is dependant on having adequate representation from groups that are historically underrepresented in political processes (Wait and Nolte, 2006).

The guideline standards are more closely aligned with Boivin et al.’s “governance discourse”, in which the values and preferences of people who access services are solicited as part of the evidence base for a policy or program (2009). The reasons that the guideline standards give for involving people who access services imply that the value of the process lies primarily in assuring a useful and high-quality end product. The pragmatic nature of guideline standards, which are intended to provide methodological guidance, may account for the narrowness of their rationale for involving people who access services.

Guideline developers working in harm reduction should be aware that guideline standards and the harm reduction literature appear to work from different assumptions about the purpose of involving people who access services. The conceptual basis for involving people who access services influences the methods perceived as appropriate, the roles and responsibilities of the individuals involved, and the measures of its effectiveness (Boivin et al., 2009; Qaseem et al., 2012). This suggests that it is not sufficient for participants in guideline development to agree that involving people who access services is important; they must also establish a shared understanding of the rationale supporting involvement.

### 4.2 Levels of involvement

Many typologies of public engagement have been described in the literature. Rowe and Frewer (2005) use a classification scheme based on the flow of information; methods in which information travels only in one direction are labelled “communication” (‘sponsor’ -> public) or “consultation” (public -> sponsor), with the term “participation” reserved for initiatives involving an exchange of information. Abelson et al. (2003) make a similar distinction, contrasting “traditional methods such as surveys, public hearings, and focus groups” with “deliberative processes” in which participants share their perspectives, reflect on their positions, and (potentially) are persuaded to change their views (p. 240). The balance of power within a deliberative process is identified as a critical determinant of its success. A much earlier attempt at classification is Arnstein's ladder of participation (1969), which distinguishes between eight categories of participation based on the degree of power granted to the population.

The guideline standards propose a variety of methods for involving people who access services. Research on guideline development shows that the methods proposed align fairly closely with those used in practice (Blackwood et al., 2020; Légaré et al., 2011). These methods are largely consultative and non-deliberative. On Arnstein's ladder of participation, most would be labelled “tokenistic”: forms of participation in which “citizens may indeed hear and be heard [but] lack the power to insure that their views will be *heeded*” (Arnstein, 1969, p. 25). The harm reduction documents, though rarely suggesting specific methods of involvement, warn against the risk of tokenism and “one-off” engagement methods (Greer et al., 2017). This discrepancy between the guideline standards and the harm reduction documents may reflect differences in the conceptual basis for involving people who access services, as discussed above.

Lower levels of involvement in the various typologies of public engagement are not invariably considered inferior. Rowe and Frewer (2005) suggest that the most appropriate type of involvement is dependant on context. The GIN Public Toolkit, which provides guidance on involving people who access services in guideline development, holds the same position: “each strategy has its strengths and limitations and their use must be tailored to specific contexts and goals” (GIN Public Working Group, 2015). Similarly, Wait & Nolte (2006) observe that the public's interest in involvement, and hence the appropriate involvement mechanism, may depend on the issue under consideration.

However, the majority of the harm reduction documents contend that consultation without any transfer of power is not meaningful. The harm reduction documents include repeated calls for shared decision-making, willingness to compromise, consideration of social and logistical factors that affect the ability of PWLLE to participate, and respect for the expertise brought by PWLLE. This does not mean that PWLLE need to be active participants in all decision-making processes, but rather that they should be the ones to decide what kind of engagement is needed (CCSA, 2021; Giacomazzo, 2021; Greer et al., 2017, 2019; Health Consumers Council Western Australia, 2016; Welsh Government, 2014). Guideline developers working in harm reduction should recognize these expectations and begin by seeking to understand how PWLLE would like to be involved.

### 4.3 Planning for involvement

As previous studies have observed, guideline standards still contain very little explicit methodological advice on involving people who access services (Selva et al., 2017). Although literature is emerging to fill this gap (GIN Public Working Group, 2015; SIGN, 2019b; Selva et al., 2017), its applicability to harm reduction may be limited by differences in the level of involvement that is perceived as appropriate.

The harm reduction literature suggests that securing adequate resources should be one of the foremost considerations in planning for the involvement of PWLLE, and repeatedly caution against underestimating the time, funding, and effort required for meaningful engagement. These topics are largely absent from the guideline standards, suggesting that guideline developers working in harm reduction should involve PWLLE in early planning and familiarize themselves with any local guidance that will help them develop a realistic budget; for instance, local payment standards for the types of work provided by PWLLE (e.g., Becu and Allan, 2017; CCSA, 2021; Pacific AIDS Network, 2018).

Securing organizational support for involving PWLLE was also perceived as critical. Approaches recommended in the harm reduction documents included training staff to facilitate involvement, potentially with the assistance of PWLLE, and creating a culture that recognizes the benefits of involving people who access services and treats it as an expected part of policy and program development (Advocacy Tasmania, 2011; AIVL, 2012; Alberta Health Services, 2018; CCSA, 2021; Welsh Government, 2014).

Although guideline standards and the harm reduction documents consistently emphasized that involving people who access services could improve the quality and acceptability of services, only a few documents noted the potential for corresponding benefits in cost-effectiveness. The difficulties of using traditional methods of analysis to quantify the economic benefits of involving people who access services are described by Pizzo et al., 2015 who observe “it is unlikely that outcomes of PPI [patient and public involvement] can be translated into the type of single monetary, effectiveness or utility measures required by traditional methods of economic evaluation, and attempts to do so would be complex and contentious” (2015, p. 1920). This may contribute to the emphasis that the harm reduction literature places on cultural change as a means of securing organizational support.

### 4.4 Limitations and reflections

This review has several limitations. Documents were screened and coded by a single author. However, results of the literature search and the coding framework were reviewed with co-authors and two groups of PWLLE to ensure completeness and validity. Our literature search was limited to documents published from 2011 to 2021 to capture contemporary material. A notable exclusion resulting from this limit is the 2008 document *Nothing About Us Without Us* (Jürgens et al., 2008). However, the key messages of this publication are captured in several of the documents included in this review.

Our search of Ovid Medline identified few relevant documents despite high recall. Fifteen of the 18 harm reduction documents included in this review were identified through the grey literature search, suggesting that experiences with involving PWLLE in developing harm reduction services are rarely published in the peer-reviewed literature. This creates challenges for researchers, as grey literature is not systematically indexed. In addition, grey literature hosted online may become inaccessible if the producing organization loses funding and ceases to have an Internet presence. Other researchers have also reported that guidance on PWLLE in service development is scarce or difficult to locate (Advocacy Tasmania, 2011; Greer et al., 2016, 2017; Ti et al., 2012). However, our intention was not necessarily to identify all publications on involving PWLLE in developing harm reduction services. Rather, we aimed to search the peer-reviewed and grey literature in a systematic manner and to describe the features of a reasonably representative sample of documents from this body of literature.

The selection of websites for the grey literature search was guided in part by the knowledge of local experts. Given the location of authors, documents from British Columbia and other Canadian provinces were more readily known and identified, leading to possible over-representation of literature from these geographic areas. We sought to offset this bias with targeted searches of the websites of relevant international organizations, as well as organizations in Australia, New Zealand, and the United Kingdom. In addition, our search of the peer-reviewed literature included a wide variety of regional terms for PWLLE.

## 5 Conclusions

Most guideline standards recognize the value of involving people who access services in guideline development. However, the extent to which guideline standards align with the harm reduction literature in their approach to involving people who access services has not previously been explored. In this study, we described the differences between guideline standards and the harm reduction literature using thematic analysis, examined the implications of these differences, and identified five essential considerations for guideline developers working in harm reduction. Our findings can be used to support the development of high-quality guidelines that align with the fundamental principles of harm reduction in their approach to involving people who access services.

The practical benefits of involving people who access services, such as improvements in the quality and acceptability of programs and policies, were affirmed in both bodies of literature. However, we found that the value of involving PWLLE in developing harm reduction services was not fully captured by guideline standards. Underlying differences in the conceptual basis for involving people who access services and a multitude of factors specific to PWLLE, such as stigma, marginalization, discrimination, and criminalization, suggest several additional considerations for guideline developers working in harm reduction. Guideline developers who apply a guideline standard without adequate consideration of the harm reduction literature may overlook the human rights argument for inclusion of PWLLE. As a result, they are likely to underestimate the importance of engagement, the required resource commitment, and the necessity of strong organizational support.

In some respects, the harm reduction literature and the guideline standards can be considered complementary. The guideline standards identify specific aspects of guideline development that benefit from involving people who access services, and the harm reduction literature provides an ethical foundation for involving PWLLE and an abundance of practical guidance. Although the guideline standards and the harm reduction literature approach involving of people who access services from different perspectives, thoughtful integration of the two paradigms can improve guideline relevancy and acceptability while empowering PWLLE.

## Contributors

AA, MF, and JB contributed to study conception and design. AA conducted the literature review, completed the preliminary analysis, and prepared the draft manuscript. AA, MF, AG, CB, KL, JM, MS, and JB contributed to critical analysis, interpretation of results, and the final manuscript. All authors approved the final article.

## Role of funding source

The sponsor had no role in study design, the collection, analysis, or interpretation of data, the writing of the report, or the decision to submit the article for publication.

## Declaration of Competing Interests

The authors declare that they have no known competing financial interests or personal relationships that could have appeared to influence the work reported in this paper.

## Appendix Supplementary materials

Image, application 1

## Footnotes

1 We recognize that the term “PWLLE”, while widely accepted in Canada, may be unfamiliar to readers from other jurisdictions. We elected to use this term because it is preferred by the two organizations of PWLLE that we consulted in the course of this research. “Peers”, though also in widespread use, is perceived as stigmatizing by some PWLLE. “People who use drugs” (PWUD) may not be an appropriate term as not all PWLLE are currently using drugs.
