# Supplementary material for: Guideline development in harm reduction: Considerations around the meaningful involvement of people who access services
Source: Drug Alcohol Depend Rep. 2022 Aug 12;4:100086. doi: 10.1016/j.dadr.2022.100086 (PMC9948926; doi:10.1016/j.dadr.2022.100086)
Supplement: Supplementary file 1 [file mmc1.docx]

**Supplementary Material**

**Appendix A . . . . . . . . . . . . . . . . . . p. 1**

**Appendix B . . . . . . . . . . . . . . . . . . p. 1**

**Appendix C . . . . . . . . . . . . . . . . . . p. 2**

**Appendix D . . . . . . . . . . . . . . . . . . p. 2**

**Supplementary Table 1 . . . . . . . . p. 4**

**Supplementary Table 2 . . . . . . . . p. 5**

**Supplementary Table 3 . . . . . . . . p. 6**

**Supplementary Table 4 . . . . . . . . p. 10**

**Supplementary Table 5 . . . . . . . . p. 15**

**Appendix A. Ovid Medline Strategy for Identifying Guidelines**

*Searched June 1, 2021. For definitions of MeSH terms (Medical Subject Headings;* ***bolded*** *in the search below), see* [*https://meshb.nlm.nih.gov/*](https://meshb.nlm.nih.gov/)*.*

Search strategy used in *Medline (Ovid MEDLINE(R) and Epub Ahead of Print, In-Process, In-Data-Review and Other Non-Indexed Citations, Daily and Versions(R) 1946 to June 01, 2021):*

(**Harm Reduction/** OR **Needle-Exchange Programs/** OR **Opiate Substitution Treatment/** OR **Naloxone/** OR ((harm reduction OR harm minimization OR harm minimisation OR needle exchange? OR needle distribution OR needle recovery OR supervised consumption OR supervised inject* OR observed consumption OR overdose prevention OR opioid substitution OR opiate substitution OR opioid agonist therapy OR opiate agonist therapy OR methadone maintenance OR methadone therapy OR suboxone OR buprenorphine OR naloxone OR narcan).ti,ab,kf)) AND (**Practice Guidelines as Topic/** OR **Guidelines as Topic/** OR guideline.pt OR guideline?.ti) AND Publication Year 2011-2021 AND Limit to English Language

**Appendix B. Grey Literature Search Strategy for Identifying Guidelines**

*Searched June 2, 2021.*

Search strategy: CADTH’S ‘Grey Matters’ checklist (‘a practical tool for searching health-related grey literature’) was used to guide the grey literature search. Sites listed under ‘Clinical Practice Guidelines’ were searched using various combinations of the following keywords:

“harm reduction” OR “harm minimization” OR “harm minimisation” OR “needle exchange” OR “needle distribution” OR “needle recovery” OR “supervised consumption” OR “supervised injection” OR “observed consumption” OR “overdose prevention” OR naloxone OR narcan OR methadone OR buprenorphine OR suboxone

Terms were modified in response to interface functionality (i.e. support for truncation, full-text search, phrase searching, etc.). Subject-specific sites (e.g BC Cancer Agency, Public Health Genomics Knowledge Base Guideline Database) were not searched unless the subject was related to harm reduction.

In addition, Google and Google Scholar were searched using the following strategy:

guideline* AND (“harm reduction” OR “harm minimization” OR “harm minimisation” OR “needle exchange” OR “needle distribution” OR “needle recovery” OR “supervised consumption” OR “supervised injection” OR “observed consumption” OR “overdose prevention” OR naloxone OR narcan OR methadone OR buprenorphine OR suboxone)

The first five pages of results were scanned for relevant publications.

**Appendix C. Ovid Medline Strategy for Identifying Best Practices for Involving PWLLE in Developing Harm Reduction Services**

*Searched Sept. 29, 2021. For definitions of MeSH terms (Medical Subject Headings;* ***bolded*** *in the search below), see* [*https://meshb.nlm.nih.gov/*](https://meshb.nlm.nih.gov/)*.*

Search strategy used in *Medline (Ovid MEDLINE(R) and Epub Ahead of Print, In-Process, In-Data-Review and Other Non-Indexed Citations, Daily and Versions(R) 1946 to Sept. 29, 2021):*

((**Harm Reduction/** OR (harm reduction OR harm minimization OR harm minimisation OR "needle exchange" or "needle distribution" or "needle recovery" or "supervised consumption" or "supervised injection" or "observed consumption" or "overdose prevention" or naloxone or narcan or methadone or buprenorphine or suboxone).ti,ab,kf)) AND ((PWLE OR PWLLE OR “lived experience” OR “living experience” OR PWUD* OR “people who use drugs” OR PWID* OR “people who inject drugs” OR PWUS* OR “people who use substances” OR peer OR peers OR drug user* OR substance user* OR service user* OR consumer* OR citizen*) AND (involv* OR participat* OR engag* OR represent*)).ti,ab,kf) AND Publication Year 2011-2021 AND Limit to English Language

**Appendix D. Grey Literature Strategy for Identifying Best Practices for Involving Service Users in Developing Harm Reduction Services**

The websites listed below were searched using browsing and variations on the following keywords: engagement, harm reduction, lived experience, peers, PWLLE, consumers, service users.

*Searched Feb. 8, 2021:*

**Harm Reduction Australia**

<https://www.harmreductionaustralia.org.au/>

**Harm Reduction International**

<https://www.hri.global/resource-library>

**International Drug Policy Consortium**

[**https://idpc.net/**](https://idpc.net/)

**New Zealand Ministry of Health – Drug publications**

<https://www.health.govt.nz/our-work/mental-health-and-addiction/addiction/alcohol-and-other-drug-policy/drug-publications>

**NZ Drug Foundation**

[**https://www.drugfoundation.org.nz/**](https://www.drugfoundation.org.nz/)

**Release**

<https://www.release.org.uk/>

**Scottish Drugs Forum**

<https://www.sdf.org.uk/>

*Searched Jun. 6, 2021:*

**ANKORS**

<https://ankors.bc.ca>

**The Australian Injecting & Illicit Drug Users’ League**

<https://aivl.org.au>

**BC Centre for Excellence in HIV/AIDS**

<http://bccfe.ca/>

**BC Harm Reduction Services**

[https://towardtheheart.com/](https://towardtheheart.com/a-z-resource-page)

**BC and Yukon Association of Drug War Survivors**

[http://bcyadws.ca/](http://bcyadws.ca/articles)

**Cactus Montreal**

<http://cactusmontreal.org/>

**CAPUD (Canadian Association of People Who Use Drugs)**

[https://www.capud.ca/](https://www.capud.ca/resources?offset=1597094611475)

**CATIE**

<https://www.catie.ca/en/prevention/substance-use>

**DUAL Ottawa**

<https://dualottawa.wordpress.com/>

**Google**

[www.google.ca](http://www.google.ca)

**Google Scholar**

[scholar.google.ca](file:///Users/ali/Documents/UBC%20Research%20Coordinator/IntJDrPo%20Revisions/Harm%20Reduction%20Doc%20Search%20Updates/Updated%20Manuscript,%20Tables,%20Data%20Extraction/scholar.google.ca)

**National Harm Reduction Coalition**

[https://harmreduction.org/](https://harmreduction.org/resource-center/)

**Open Society Foundations**

<https://www.opensocietyfoundations.org/>

**Pacific AIDS Network**

<https://pacificaidsnetwork.org/>

**SOLID Outreach**

<https://solidvictoria.org>

**VANDU (Vancouver Area Network of Drug Users)**

<https://vandureplace.wordpress.com/>

**Western Aboriginal Harm Reduction Society**

<https://wahrs.ca>

| **Supplementary Table 1. Guideline standards identified in harm reduction guidelines and/or accounts of their development.** | |
| --- | --- |
| **Document** | **Guideline standard(s) identified** |
| Atkinson, T. J., Pisansky, A. J. B., Miller, K. L., & Yong, R. J. (2019). Common elements in opioid use disorder guidelines for buprenorphine prescribing.*The American Journal of Managed Care, 25*(3), e88-e97. | No standard identified. |
| Blandthorn, J., Bowman, E., Leung, L., Bonomo, Y., & Dietze, P. (2018). Managing opioid overdose in pregnancy with take‐home naloxone.*Australian & New Zealand Journal of Obstetrics & Gynaecology, 58*(4), 460-462. | No standard identified. |
| British Columbia Centre on Substance Use. (n.d.). Implementing supervised consumption services – operational guidance. BC Ministry of Health. | No standard identified. |
| College of Physicians and Surgeons of British Columbia. (February 2014; updated September 2015*). Methadone maintenance program: Clinical practice guideline.* | No standard identified. |
| Fairbairn, N., Ross, J., Trew, M., Meador, K., Turnbull, J., MacDonald, S., Oviedo-Joekes, E., Le Foll, B., Goyer, M., Perreault, M., & Sutherland, C. (2019). Injectable opioid agonist treatment for opioid use disorder: A national clinical guideline.*Canadian Medical Association Journal (CMAJ), 191*(38), E1049-E1056. | AGREE II  GRADE |
| Farmer, C. M., Lindsay, D., Williams, J., Ayers, A., Schuster, J., Cilia, A., Flaherty, M. T., Mandell, T., Gordon, A. J., & Stein, B. D. (2015). Practice guidance for buprenorphine for the treatment of opioid use disorders: Results of an Expert panel process.*Substance Abuse, 36*(2), 209-216. | No standard identified. |
| Handford, & Scholars Portal Books: Legislative Library of Ontario. (2011). *Buprenorphine/naloxone for opioid dependence: Clinical practice guideline*. CAMH. | Canadian Task Force on Preventive Health Care |
| Korownyk, C., Perry, D., Ton, J., Kolber, M. R., Garrison, S., Thomas, B., Allan, G. M., Bateman, C., de Queiroz, R., Kennedy, D., Lamba, W., Marlinga, J., Mogus, T., Nickonchuk, T., Orrantia, E., Reich, K., Wong, N., Dugré, N., & Lindblad, A. J. (2019). Managing opioid use disorder in primary care: PEER simplified guideline.*Canadian Family Physician, 65*(5), 321-330. | GRADE |
| Registered Nurses’ Association of Ontario. (March 2015). Engaging clients who use substances. International Affairs & Best Practice Guidelines. | AGREE II  SIGN 50 |
| Registered Nurses’ Association of Ontario. (February 2018). Implementing supervised injection services. International Affairs & Best Practice Guidelines. | AGREE II  SIGN 50 |
| Scheibe, A., Sibeko, G., Shelly, S., Rossouw, T., Zishiri, V., & Venter, W. D. F. (2020). Southern African HIV clinicians society guidelines for harm reduction.*Southern African Journal of HIV Medicine, 21*(1), 1161-1161. | GRADE^1^ |
| Schlesinger, E. B., Geminn, W., Hohmeier, K. C., & Burley, J., Howard L. (2018). Development and implementation of Tennessee nonresidential buprenorphine treatment guidelines.*Innovations in Pharmacy, 9*(3), 1-4. | No standard identified. |
| Supervised Injectable Opioid Agonist Treatment Guidance Committee (B.C.), British Columbia Centre on Substance Use, British Columbia. Ministry of Health, & British Columbia Government EBook Collection. (2017). *Guidance for injectable opioid agonist treatment for opioid use disorder*. Ministry of Health. | No standard identified. |
| Tsuyuki, R. T., Arora, V., Barnes, M., Beazely, M. A., Boivin, M., Christofides, A., Patel, H., Laroche, J., Sihota, A., & So, R. (2020). Canadian national consensus guidelines for naloxone prescribing by pharmacists.*Canadian Pharmacists Journal, 153*(6), 347-351 | No standard identified. |
| NICE. (26 March 2014). Needle and syringe programmes (PH52). Public health guideline. www.nice.org.uk/guidance/ph52 | NICE |
| NICE. (2020). Smoking: harm reduction (PH45). Public health guideline. www.nice.org.uk/guidance/ph45 | NICE |
| ^1^ This document was described as being “informed by a review of evidence and guidance from the World Health Organization” (Scheibe et al., 2020, p. 3). The WHO uses GRADE for guideline development (WHO Handbook for Guideline Development, 2012). | |

| **Supplementary Table 2: Additional details of guideline standards.** | | |
| --- | --- | --- |
| **Guideline Standard** | **Purpose** | **Target Audience** |
| AGREE Next Steps Consortium, 2017 | ‘The purpose of the AGREE II, isto provide a framework to: 1. Assess the quality of guidelines; 2. Provide a methodological strategy for the development of guidelines; an3. Inform what information and how information ought to be reported in guidelines.’ (p. 0) | Health care providers, guideline developers, policy makers, and educators (p. 3) |
| Institute of Medicine, 2011 | ‘[. . .] the Institute of Medicine undertook this study to develop a set of standards for developing rigorous, trustworthy clinical practice guidelines’ (p. ix) | Not explicitly identified, but states ‘The committee recommends that CPG developers adhere to these standards, and that CPG users adopt CPGs compliant with these standards’ (p. 109) |
| NICE, 2020 | ‘This manual explains the processes and methods NICE uses for developing, maintaining and updating NICE guidelines’ (p. 13) | ‘[This manual] is primarily for: NICE staff involved in developing guidelines; NICE contractors [. . .] members of the committees that develop the guidelines [. . .] It is also likely to be of interest to a broader audience, including developers of other guidance, stakeholders and users of NICE guidelines.’ (p. 12-13) |
| Schünemann et al., 2013 [GRADE Handbook] | ‘The handbook is intended to be used as a guide by those responsible for using the GRADE approach to produce GRADE's output, which includes evidence summaries and graded recommendations.’ (p.3) | ‘Systematic review and health technology assessment (HTA) authors, guideline panelists and methodologists who provide support for guideline panels.’ (p. 3) |
| Scottish Intercollegiate Guidelines Network (SIGN), 2019 | ‘The principal aim of this manual is to provide a reference tool that may be used by individual members of guideline development groups as they work through the development process [. . .] A secondary aim of this manual is to be transparent about the methods used to develop SIGN guidelines, and to instil confidence that the potential biases of guideline development have been addressed adequately, and that the recommendations are both internally and externally valid, and feasible for practice..’ (p. 1) | Members of guideline development groups (p. 1). |
| Qaseeem et al., 2012 [Guidelines International Network] | ‘This article presents G-I-N’s proposed set of key components for guideline development [. . .] It is hoped that this article promotes discussion and eventual agreement on a set of international standards for guideline development.’ (p. 525) | Not explicitly identified, but states ‘the proposed key components presented in this article should help guideline developers and users assess the strengths and weaknesses of a guideline’ (p. 528). |

| **Supplementary Table 3. Additional details of harm reduction documents.** | | |
| --- | --- | --- |
| **Harm Reduction Documents** | **Purpose** | **Definition of Involving People Who Access Services** |
| Advocacy Tasmania, 2011 | ‘to establish a consumer participation framework to ensure that consumers have  input into the planning, development and delivery of ATOD [Alcohol and Other Drug Treatment] services in Tasmania.’ (p. 4) | ‘Consumer Engagement: In this context, consumer engagement implies that there is a process with which the consumer is involved, and that this process is active. It involves a relationship between two parties, the consumer and the service (or the broader service system) and that both parties contribute to the process. According to this definition, processes during which the consumer is a passive recipient, while appropriate and helpful in many circumstances, are not technically classed as consumer engagement. However, such information providing processes can be a catalyst for consumer engagement in the longer term and are important to consider  within this framework.’ (p. 6) |
| Alberta Health Services, 2018 | ‘This framework is intended to provide guidance, considerations and recommendations to teams, zones, sites, programs and clinics to support meaningful and respectful engagement practices’ (p. 6) | ‘This framework is based on and adapted from a multi-dimensional model for patient and family engagement in health and healthcare developed by Carmen et al, 2013 (Figure 2). This model is linked to a public participation spectrum (International Association for Public Participation – _IAP2) that frames a continuum of engagement across five levels identified as; inform, consult, involve, collaborate, and empower.’ (p. 11) |
| Australian Injecting & Illicit Drug Users League, 2012 | ‘To document the contributions of drug user organisations, and people who use illicit drugs, to the development of drug policy in Australia over the last 20 years; 2. ‘To identify key principles that underlie the meaningful engagement of people who use illicit drugs and peer-based drug user organisations in drug policy activity; and 3. To identify barriers and facilitators of meaningful engagement, such that people whose role it is  to develop drugs policy, primarily in government agencies, are able to make more informed policy  decisions.’ (p. 14) | ‘Here, consumer participation is broadly defined as ‘the process of involving health consumers in decision making about health service planning, policy development, setting priorities and quality issues in the delivery of health services’.^14^ In more ideological terms it can also be defined as ‘more sharing, not only of information and opinion, but also of decision making power. Real participation means joint problem-solving, joint decision-making, joint responsibility.’^15^ (p. 8) |
| Belle-Isle et al., 2016 | ‘to provide ‘evidence-based practice guidelines for allies to better include people who use drugs at their decision-making tables.’ (p. 1) | No definition identified. |
| Canadian AIDS Society, 2015 | ‘to guide those who wish to get involved locally and mobilize to improve the life conditions of people who use drugs in their area [. . .] [to provide] advice on how to include people who use drugs in decisions that affect their lives and tips to assist in building capacity to respond to the needs of people who use drugs.’ (p. 2) | No defnition identified. |
| Canadian Association of People Who Use Drugs, 2014 | This is the final report of a national meeting to ‘Strengthen a radical national movement; Share current work on principles of self-representation in harm reduction, HIV/AIDS and substance use research, policy, and programming; Develop guidelines for meaningful participation of people who use[d] illicit drugs in the development of drug policy, harm reduction and HIV/AIDS policy, services, and research.’ (title page). | No defnition identified. |
| Canadian Centre on Substance Use and Addiction, 2021 | to ‘consolidate[s] the evidence and knowledge available for working with groups of people with lived and living experience and their families and friends (LLEAFF groups) to ensure that those engaging with them follow best practices.’ (p. 2) | No definition identified. |
| Giacomazzo, 2021 | ‘This article summarizes available literature and discusses the importance of the meaningful engagement of people who use drugs, sometimes — and controversially — called “peers,” in harm reduction programs. This includes information on the roles that people who use drugs take on in programming, the advantages of their engagement, and barriers and facilitators to their engagement.’ (para. 1) | ‘The meaningful engagement of people who use drugs has several facets: tokenization is avoided;^5,6^ community members participate in and/or lead the design, implementation and evaluation of programs;^7^ and people who use drugs are included in decision-making processes.^6^ Meaningful engagement also means that people who use drugs choose whether they want to participate, how they are represented and how they engage.^7’^ |
| Greer et al., 2019 | ‘The purpose of this study was to explore perspectives of PE [peer engagement] among PWUD across BC to identify barriers and enablers to PE.’ (p. 228) | ‘‘Engagement’, ‘involvement,’ and ‘participation’ are terms that have been used in the literature, but active, empowering, and transformative PE [peer engagement] ultimately depends on the level of commitment and active participation in the decision-making process (Ocloo and Matthews 2016; Cornwall and Brock 2005; Leal 2007). In the current paper, engagement signifies a commitment to the PE process but is not necessarily action-based. In other words, PE may fall short in that individuals may be engaged but not enabled to actively participate.’ (p. 228) |
| Greer et al., 2016 | ‘This paper aims to describe and evaluate the peer engagement efforts undertaken by the BCHRSS [British Columbia Harm Reduction Strategies and Services] committee from 2010 to 2014. We highlight key lessons learned  and improvements needed to ensure meaningful peer engagement in the planning, delivery and evaluation of  harm reduction efforts.’ (p. 2) | ‘Peer engagement has been defined as a community-based approach to decision making by “consulting and collaborating with decision makers using a bottom-up  approach in order to better address the needs of the community” [7].’ (p. 2) |
| Greer et al., 2017 | ‘This document provides both an overview and details of these [peer engagement practices] to support meaningful and equitable engagement between service providers and peers’ (p. 6) | ‘Peer engagement can be defined as the active participation of people with lived experience of substance use in different research, program, and policy decision-making processes.’ (p. 5) |
| Health Consumers Council Western Australia, 2016 | ‘To identify ‘six principles developed [to engage and support consumers to participate at a range of levels in the AOD [Alcohol and Other Drugs] sector], as well as stragies to engage and support consumers to participate at a range of levels in the AOD sector in WA [Western Australia]’ (p. 6) | ‘Participation and engagement are used interchangeably throughout this document. Consumer engagement or participation refers to any activity that incorporates consumer aspirations and needs into decision making at an individual, service, sector and systems level or ‘Participation occurs when consumers, carers and community members aremeaningfully involved in decision-making about health policy and planning, care and treatment, and the well-being of themselves and the community’ (cited in Consumer participation in the Australian alcohol and other drug sector; ANCD page 13)’ (p. 4) |
| Kools, 2013 | “This brochure highlights some of the examples of excellent practices in peer involvement in Europe.” (p. 3) | “As a working definition for peer work, and describing main aspects, we will use the following description: “Peer work is a freestanding initiative or a collaboration between community members and an agency, aiming at meaningful involvement of peers and based on principles of mutuality and empowerment” [. . .] three examples of good meaningful involvement are be distinguished: 1. people who use drugs participating in decision-making about programmes and services; 2. recognising and respecting the ex- pertise of drug users who know about drug-use patterns; 3. people who use drugs implementing and providing services or programmes as peer educators, evaluators or programmers.” (p. 1, 3) |
| NSW Ministry of Health, 2019 | ‘Goal of the AOD [Alcohol and Other Drugs] Consumer Engagement Framework: People with a lived experience actively working together with the AOD Branch to improve the experiences of customers accessing health services who are impacted by alcohol and other drug use through these guiding principles: Partnership, Humanity: People First, Diversity,  Meaningful engagement, Participation; By applying these engagement approaches: Inform, Consult, Involve, Collaborate, Embower and embed; To achieve these shared objectives: Person centred care; Barriers are identfied, addressed, and overcome; Empowerment; Consumer participation is embedded and respected in all AOD work; Open communication.’ (p. 5) | No definition identified. |
| Schiffer & C-EHRN, 2021 | “This paper aims to identify barriers and facilitators during the participation process, by analysing various determinants during the implementation process. Based on this analysis, a more specific approach will be suggested, building on a number of existing participation models and theories.” (p. 8) | No definition identified. |
| Ti et al., 2012 | ‘to provide a summary of the available evidence on peer engagement among PWUD and its role in policy and program development. Findings from this review will identify gaps in the literature as well as provide important information on how to more effectively engage peers in policy and program making decisions.’ (p. 2) | ‘Peer engagement is a community-based approach and we have defined it as the process of consulting and collaborating with decision makers using a bottom-up approach in order to better address the needs of the community’ (p. 2) |
| UNODC, 2017 | ‘This tool offers practical advice on how to implement these programmes and these approaches [effective HIV and HCV prevention interventions for people who inject drugs, in the context of harm reduc­tion15 and HIV prevention for key populations] for and with people who inject drugs, across the full continuum of HIV and HCV prevention, diagnosis, treatment and care’ [. . .] Chapter 4: Service Delivery Approaches describes how to design services and how community members can be involved in varying aspects of service delivery, including (but not limited to) outreach to people who inject drugs, peer navigation and running drop-in centres. (p. xvii, xxi) | No definition identified. |
| Welsh Government, 2014 | ‘to provide guidance on the involvement of service users to Area Planning Boards (APBs), service commissioners, planners and providers and to service users, their families and carers [. . .] to promote the benefits of involving service users in the development of policy, and in the design, planning, delivery and evaluation of substance misuse services, at all levels’ (p. 2, 5) | ‘The term ‘involvement’ is the focus of this guidance and is used throughout. The term is used in a broad sense to cover both active, ongoing joint working and true and full participation (actively taking part in) and the consultation process (looked to for information and advice). However ‘involvement’ means working towards and aspiring to full participation.’ (p. 23) |

| **Supplementary Table 4. Codebook used in thematic analysis.** | | |
| --- | --- | --- |
| **THEME 1: REASONS FOR INVOLVEMENT*.*** *Includes statements that directly or indirectly acknowledged the value of involvement of people who access services in service development; statements identifying specific areas of policy and programming that would be influenced by involvement; statements on the consequences or implications of inadequate involvement.* | | |
| **Subtheme** | **Description** | **Example** |
| Quality | Involvement of people who access services as a means of improving the overall balance, comprehensiveness, or quality of policy and program development | “The [guideline] draft is also sent to at least two lay reviewers in order to obtain comments from the patient’s perspective. Reviewers are asked to comment primarily on the comprehensiveness and accuracy of interpretation of the evidence base supporting the recommendations in the guideline.” (Scottish Intercollegiate Guidelines Network (SIGN), 2019) |
| Acceptability | Involvement of people who access services as a means of increasing the acceptability of policy or programming, improving uptake, or facilitating a sense of ownership | “For a recommendation to be implemented effectively, it is important that the outcomes are sufficiently valued by patients for them to be willing to adhere to the treatment.” (Scottish Intercollegiate Guidelines Network (SIGN), 2019) |
| Equity | Value of involvement of people who access services in increasing health equity, either directly or indirectly | “As the reviewer develops themes from [the literature search on patient issues], they will pay particular attention to anything that suggests there are population groups that are disadvantaged and ensure their interests are specifically considered by the guideline development group.” (Scottish Intercollegiate Guidelines Network (SIGN), 2019) |
| Human rights | Involvement of people who access services as a moral imperative; right of people who access services to be involved in decisions that affect their lives. | “Peers have an ethical and imperative right to be involved in the decisions affecting their lives and often, they are the ones who are the most knowledgeable on how to most effectively approach their population.” (Ti et al., 2012) |
| Value to participants | Personal benefits of involvement for people who access services and other participants | “In addition, through dialogue and critical reflection, it is theorized that everyone at the table will experience a shift in consciousness. This shift can change how we see ourselves and each other as well as the balance of power at the table.” (Belle-Isle et al., 2016) |
| Scope | Value of involving people who access services in identifying issues relevant to people who access services; value of involvement in defining scope of services | “One should seek evidence relating to all patient-important outcomes and for the values patients place on these outcomes as well as related management options.” (Schünemann et al., 2013) |
| Recommendations | Value of involvement of people who access services in informing the direction and strength of guideline recommendations | “The greater the variability in values and preferences, or uncertainty about typical values and preferences, the more likely a weak recommendation is warranted.” (Schünemann et al., 2013) |
| Credibility | Use of involvement of people who access services to give guideline credibility | “First, as a matter of transparency, detailed in preceding content, the involvement of one or more consumer representatives provides a window into the process and some assurance that guidelines were not developed “behind closed doors” to suit special interests other than theirs.” (Institute of Medicine, 2011) |
| Cost-effectiveness | Use of involvement of people who access services to reduce costs | “[. . .] while consumer engagement can at times present additional costs to organisations, feedback from consumers may also lead to cost savings. For example, if consumers identify that a particular component of a service is not helpful, out-dated, or no longer relevant, this may in fact help agencies and system managers to identify more cost effective way to deliver a service.” (Advocacy Tasmania, 2011) |
| **THEME 2: METHODS OF INVOLVEMENT.** *Includes descriptions of specific methods of involving people who access services.* | | |
| **Subtheme** | **Description** | **Example** |
| Leveraging & supporting communities of people who access services | Working with communities of people who access services, including organizations of PWLLE; supporting these communities in capacity building | “11th Recommendation: Support organizations of people who use drugs to support inclusion. To support the inclusion of people who use drugs, it must be acknowledged that there are organisations of people who use drugs across Canada that have a mandate of inclusion. We are there to help you work with us. That is why our 11th recommendation is: Support organizations of people who use drugs to support inclusion.” (Canadian AIDS Society, 2015) |
| Committee membership | Involving people who access services as members of committees working on policy or program development | “There are various methods for ensuring that these perspectives inform the different stages of guideline development by stakeholders. For example, formal consultations with patients/public to determine priority topics, participation of these stakeholders on the guideline development group, or external review by these stakeholders on draft documents.” (AGREE Next Steps Consortium, 2017) |
| Interviews & focus groups | Interviews, focus groups, or invitation-only workshops as recommended method of involving people who access services | “Alternatively, information could be obtained from interviews of these stakeholders or from literature reviews of patient/public values, preferences or experiences.” (AGREE Next Steps Consortium, 2017) |
| Public forums | Holding public meetings or forums to provide all members of the public with the opportunity to view or contribute to policy or program development | “Further patient and public participation in guideline development is achieved by involving patients, service users, carers and voluntary organisation representatives at the national open meeting which is held to discuss each draft guideline.” (Scottish Intercollegiate Guidelines Network (SIGN), 2019) |
| Literature review | Using literature review or existing studies to determine views of people who access services | “These judgments are ideally informed by a systematic review of the literature focusing on what the target population considers as critical or important outcomes for decision making.” (SchIN ZOTERO_ITEM CSL_CI |
| Consultations | Consultation (written, oral, or unspecified) as recommended method of involving people who access services; inviting selected people who access services to provide written or oral testimony to policy or program developers | “[. . .] For example, formal consultations with patients/public to determine priority topics.” (AGREE Next Steps Consortium, 2017) |
| Review of drafts | Circulating material to people who access services for review | “A draft of the CPG at the external review stage or immediately following it (i.e., prior to the final draft) should be made available to the general public for comment. Reasonable notice of impending publication should be provided to interested public stakeholders.” (Institute of Medicine, 2011) |
| Engagement at point of care | Development of policies that support shared decision-making (provider and person accessing services) at the point of care | “A weak recommendation implies that not all individuals will be best served by the recommended course of action. There is a need to consider more carefully than usual the individual patient’s circumstances, preferences, and values. When there are weak recommendations caregivers need to allocate more time to shared decision making, making sure that they clearly and comprehensively explain the potential benefits and harms to a patient.” (Schünemann et al., 2013) |
| Clinicians as representatives of people who access services | Using clinicians, guideline developers, or members of the general public as proxies for people who access services (i.e. soliciting their views on what people who access services would want) | “In the **absence of such evidence**, panel members should use their prior experiences with the target population to assume the relevant values and preferences.” (Schünemann et al., 2013) |
| **THEME 3: FACTORS IN SUCCESS.** *Includes descriptions of barriers, facilitators, and practical aspects of involvement of people who access services.* | | |
| **Subtheme** | **Description** | **Example** |
| Resources | Resources required for involvement of people who access services, including considerations around finances and time | “Although certain standards, such as those directed to patient and public involvement in the CPG development process and external review, may appear particularly resource intensive, strategies to increase effective public participation can minimize this burden.” (Institute of Medicine, 2011) |
| Logistics | Practical considerations for involvement of people who access services, including transportation, modes of communication, access to substances, location, and childcare | “If you want us to travel please: DO help with arranging methadone carries; DO arrange for advice from a local person who uses drugs – drugs may be more dangerous in a different city and travelling puts us at risk; DO provide accommodation close to the meeting space; DO have a healthcare provider available to support us; DON’T invite us at the last minute and assume we can deal with this alone; DON’T just leave us on our own in cities we don’t know; DON’T assume we have identification (or credit cards) to check into hotels or board flights.” (Greer et al., 2017) |
| Lack of research | The need for systematic and evidence-based methods for involvement of people who access services; the lack of research on this topic | “Because frameworks for consumer involvement are based on limited practical experience (Bastian, 1996; Duff et al., 1993), there is little consensus about how and when to involve consumers and what to expect from them during guideline development (van Wersch and Eccles, 1999).” (Institute of Medicine, 2011) |
| Representation | The challenge of selecting individuals or group to represent views of entire population; ways of addressing this problem | “Well resourced guideline panels will usually complement such studies with consultation with individual patients and patients’ groups. The panel should discuss whose values these people represent, namely representative patients, a defined subset of patients, or representatives of the general population” (Schünemann et al., 2013) |
| Skills and literacy | Limited scientific literacy and/or lack of required skills and objectivity as barriers to involvement of people who access services; ways of addressing this challenge | “[. . .] involvement of laypersons in practice guideline development may be problematic, particularly if they lack relevant training and scientific literacy [. . .] A second challenge occurs when a consumer representative has a personal experience with the disease or an advocacy role interfering with the ability to examine evidence and recommendations dispassionately.” (Institute of Medicine, 2011) |
| Transparency | The need to ensure clear expectations around the nature and extent of involvement of people who access services; the need for transparency around opportunities for involvement of people who access services and the incorporation of findings from involvement of people who access services into policy and programming; the need to openly evaluate the process | “Whatever the source of estimates of typical values and preferences, explicit, transparent statements of the panel’s choices are imperative (see 6.3.3 Providing transparent statements about assumed values and preferences).” (Schünemann et al., 2013) |
| Trust and power | Trust and power in involvement of people who access services; the role of mutual respect and open communication | “Including peers at decision-making tables should, in theory, create equal and distributed power and voices at the table; thus, creating more equitable and fair policies for communities that are often silenced (22). However, people who use drugs are often affected by health and social inequities that position them with less power and resources due to economic, social, historical, and political conditions in society. These conditions that peers experience in our society create inequitable power relations with decision makers and other members of the public.” (Greer et al., 2017) |
| Compensation | Providing compensation for people who access services who are involved in program and policy development. | “Consideration must be given to the payment of service users and the re-imbursement of any expenses. Expenses should be offered, rather than sought by service users and payments should be made on the day of the event or, if possible, in advance.” (Welsh Government, 2014) |
| Stigma | Negative perceptions of people who access services as a barrier; discrimination or fear of discrimination as a barrier | “Barriers of stigma and discrimination may have made it more difficult for policy makers to appreciate the benefits of involving peers in policy decisions [. . .] Such negative attitudes often impeded participation of PWUD in treatment decision making by undermining their self-worth and self-confidence.” (Ti et al., 2012) |
| Participant capacity | The need to consider and accommodate individual factors influencing participation in efforts to involve people who access services. | “A lack of self-efficacy, instability, and competing priorities made engagement challenging. Many discussed being “too busy, doing what you gotta do” [. . .] Participants suggested that it was hard to engage because it meant altering their routine, which they were unwilling to do without consistent and sustainable PE opportunities.” (Greer et al., 2019) |
| Tokenism | The risk of inauthentic involvement of people who access services (i.e. involvement for the sake of appearances; involvement without sharing decision-making power) | “If you want to include us, it is important to do it fully. There is nothing more frustrating than having the impression that we are there as tokens and that our opinion is not sought, not listened to and not taken seriously.” (Canadian AIDS Society, 2015) |
| Social support | The value of social support for people who access services who are involved in policy or program development | “Finally, having two of us attend meetings together will make us feel more at ease with other participants.” (Canadian AIDS Society, 2015) |
| Systemic factors | The impact of legal environment, political environment, and organizational culture on involvement of people who access services | “It was also felt that external political agendas often drive drugs policy. According to one respondent, “unfortunately, it seems that most drug policies are either influenced more by political concerns than by drug user organisations and drug users at this stage”.” (Australian Injecting & Illicit Drug Users League, 2012) |

| **Supplementary Table 5. Key to Tables 3-5.** | | |
| --- | --- | --- |
| **Document Type** | **No.** | **Reference** |
| Harm Reduction Literature | 1 | Advocacy Tasmania, 2011 |
|  | 2 | Alberta Health Services, 2018 |
|  | 3 | Australian Injecting & Illicit Drug Users League, 2012 |
|  | 4 | Belle-Isle et al., 2016 |
|  | 5 | Canadian AIDS Society, 2015 |
|  | 6 | Canadian Association of People Who Use Drugs, 2014 |
|  | 7 | Canadian Centre on Substance Use and Addiction, 2021 |
|  | 8 | Giacomazzo, 2021 |
|  | 9 | Greer et al., 2019 |
|  | 10 | Greer et al., 2016 |
|  | 11 | Greer et al., 2017 |
|  | 12 | Health Consumers Council Western Australia, 2016 |
|  | 13 | United Nations Office on Drugs and Crime, 2017 |
|  | 14 | New South Wales Ministry of Health, 2019 |
|  | 15 | Ti et al., 2012 |
|  | 16 | Welsh Government, 2014 |
|  | 17 | Kools, 2013 |
|  | 18 | Schiffer & Correlation-European Harm Reduction Network, 2021 |
| Guideline Standards | 1 | Schünemann et al., 2013 |
|  | 2 | Scottish Intercollegiate Guidelines Network, 2019 |
|  | 3 | AGREE Next Steps Consortium, 2017 |
|  | 4 | NICE, 2020 |
|  | 5 | Institute of Medicine et al., 2011 |
|  | 6 | Qaseem et al., 2012 |
